# Evaluation of World Health Organization–Recommended Hand Hygiene Formulations

**DOI:** 10.3201/eid2609.201761

**Published:** 2020-09

**Authors:** Miranda Suchomel, Maren Eggers, Steffen Maier, Axel Kramer, Stephanie J. Dancer, Didier Pittet

**Affiliations:** Medical University, Vienna, Austria (M. Suchomel, S. Maier);; Laboratory Prof. Gisela Enders & Colleagues MVZ and Institute of Virology, Infectiology and Epidemiology e.V., Stuttgart, Germany (M. Eggers);; University of Medicine, Greifswald, Germany (A. Kramer);; Hairmyres Hospital, National Health Service, Lanarkshire, Scotland, UK (S.J. Dancer);; Edinburgh Napier University, Edinburgh, Scotland, UK (S.J. Dancer);; World Health Organization, Geneva, Switzerland (D. Pittet);; University of Geneva Hospitals and Faculty of Medicine, Geneva (D. Pittet)

**Keywords:** 2019 novel coronavirus disease, coronavirus disease, COVID-19, severe acute respiratory syndrome coronavirus 2, SARS-CoV-2, viruses, respiratory infections, zoonoses, World Health Organization, WHO formulation, modified, hand hygiene, surgical hand preparation, alcohol-based hand rub, hand sanitizer, glycerol

## Abstract

As a result of the coronavirus disease pandemic, commercial hand hygiene products have become scarce and World Health Organization (WHO) alcohol-based hand rub formulations containing ethanol or isopropanol are being produced for hospitals worldwide. Neither WHO formulation meets European Norm 12791, the basis for approval as a surgical hand preparation, nor satisfies European Norm 1500, the basis for approval as a hygienic hand rub. We evaluated the efficacy of modified formulations with alcohol concentrations in mass instead of volume percentage and glycerol concentrations of 0.5% instead of 1.45%. Both modified formulations met standard requirements for a 3-minute surgical hand preparation, the usual duration of surgical hand treatment in most hospitals in Europe. Contrary to the originally proposed WHO hand rub formulations, both modified formulations are appropriate for surgical hand preparation after 3 minutes when alcohol concentrations of 80% wt/wt ethanol or 75% wt/wt isopropanol along with reduced glycerol concentration (0.5%) are used.

Because commercial products are hardly or no longer available due to the coronavirus disease (COVID-19) pandemic, alcohol-based hand rub formulations for hygienic and surgical hand treatment published by the World Health Organization (WHO) in 2009 (*1*) for local production in developing countries are now being produced for use in hospitals worldwide. As shown previously (*2*), neither the formulation based on ethanol 80% vol/vol (WHO I) nor that based on isopropanol 75% vol/vol (WHO II), meets the efficacy requirements of the European Norm (EN) 12791 (*3*), which must be fulfilled to obtain approval as a surgical hand preparation in Europe. Each WHO-recommended formulation is also insufficient for hygienic hand antisepsis when 3 mL is applied for 30 seconds (*4*) according to the test method described in EN 1500 (*5*). The requirements can be met only if the volume is doubled (6 mL) and exposure is extended to 60 seconds (*4*). But sufficient efficacy has been achieved by using modified WHO formulations with an increased alcohol concentration of 80% wt/wt ethanol or 75% wt/wt isopropanol at 3 mL for 30 seconds (*4*). 

On the basis of those results, we modified both WHO formulations by increasing their alcohol concentrations through changing their volume percentages into weight percentages and by prolonging the duration of application from 3 to 5 minutes. These modifications have been shown to render the immediate effects of both formulations noninferior to the reference of EN 12791, but this improvement was not observed for the so-called 3-hour effect (i.e., 3 hours after hand antisepsis) (*6*). Because the high glycerol concentration (1.45% vol/vol) of the original formulations has been shown to exert a negative influence on the 3-hour efficacy of alcohols (*7*), we performed further studies by reducing the glycerol content of the WHO formulations by 50%. By increasing the alcohol concentration by ≈5% and reducing glycerol concentrations to 0.725%, both modified WHO formulations meet the efficacy requirements of EN 12791 when used for 5 minutes (*8*). Although both new formulations were successfully tested for a 5-minute application, our suggestions for improving efficacy were not accepted by the WHO because the common duration for surgical hand preparation in most hospitals is 3 minutes. Furthermore, no information on dermal tolerability and healthcare workers’ acceptance of these modified formulations was available.

In 2019, Menegueti et al (*9*) showed that a modified WHO I formulation containing only 0.5% glycerol led to better ratings of skin tolerance than the original WHO formulation containing 1.45% or a modification containing 0.75% glycerol. Because all such alternative formulations require testing for not only dermal tolerability but also for bactericidal performance, we investigated the efficacy of these modified WHO formulations (mass instead of volume percentage ethanol or isopropanol and 0.5% instead of 1.45% glycerol) according to EN 12791 (*3*), with an application duration of 3 minutes, as commonly used in surgical theaters in Europe.

## Materials and Methods

We used 2 formulations in this study. WHO I modified comprised ethanol (for analysis; Merck KGaA, https://www.emdgroup.com) 80% wt/wt, hydrogen peroxide (for analysis; Merck) 0.125% vol/vol, and glycerol (for analysis; Merck) 0.5% vol/vol. WHO II modified comprised isopropanol (for analysis; Merck) 75% wt/wt, hydrogen peroxide 0.125% vol/vol, and glycerol 0.5% vol/vol. For the reference alcohol of EN 12791, we used N-propanol (for analysis; Merck) 60% (vol/vol) without additions (*3*).

We recruited 24 volunteers from the Institute for Hygiene and Applied Immunology, Medical University of Vienna (Vienna, Austria), to participate in the study. Exclusion criteria were age <18 years and skin breaks on hands (e.g., cuts, abrasions or other skin disorders). Nails were short and clean and volunteers agreed to not use any antibacterial soap or other antibacterial substance during the trial, starting from 1 week before testing. Volunteers were also asked to not use any hand rub or hand cream on trial days. All volunteers provided written informed consent. The study protocol was approved by the institutional ethics committee of the Medical University of Vienna (ethical vote no. 2092/2019).

Culture media were as described in EN 12791 (*3*). For sampling and dilution fluids, we used tryptic soy broth (CASO broth; Merck). For counting plates, we used tryptic soy agar (CASO agar; Merck). Neutralizing agents were not necessary for any of the tested modified WHO formulations because even dilution in pure broth without supplement in previous validation tests has been shown to neutralize any antimicrobial effect (*4*).

We compared the efficacy of the modified WHO formulations with that of the standardized reference surgical hand treatment described in EN 12791. We used a Latin-square design with 3 groups, each with 8 randomly allocated volunteers, and as many experimental runs as there were formulations, including the reference. In every run, we tested all hand treatment procedures concurrently. At the end of the third test run, every volunteer had used each formulation once. We spaced test runs apart by 1 week to allow reversion of normal skin flora.

We used the test method described in EN 12791 (*3*). In brief, after a preparatory hand wash for 1 minute with 5 mL of 20% nonmedicated soap applied onto wet hands to remove transient bacterial flora and any other soil, participants rinsed their hands under running tap water and dried them with soft paper towels. Pretreatment values were established by rubbing and kneading the fingertips, including the thumbs, of both hands for 1 minute at the base of a petri dish (diameter 9 cm) containing 10 mL of sampling fluid, one for each hand. Subsequently, surgical hand antisepsis was performed according to the standardized hand rub procedure of EN 12791 by applying and rubbing as many 3-mL portions of the study formulations (i.e., WHO I modified, WHO II modified, or reference) onto both hands up to the wrists as necessary to keep the hands wet for 3 minutes. 

According to EN 12791, the efficacy of a preoperative hand procedure is determined immediately and 3 hours after hand antisepsis. Thus, to assess the posttreatment values of a formulation, we sampled one randomly selected hand as described for the pretreatment values immediately after hand antisepsis (immediate effect). The other hand was gloved and sampled 3 hours later to assess the 3-hour effect. We performed quantitative surface cultures from all sampling fluids and dilutions on tryptic soy agar, incubated counting plates for a total of 48 hours at 36°C ± 1°, and counted colony-forming units. 

For statistical analyses, we expressed all colony counts per mL of sampling fluid as decadic logarithms_._ From the intra-individual differences between log_10_ pretreatment minus log_10_ posttreatment values, we calculated individual log_10_ reduction factors separately for immediate and 3-hour effects. We tested pretreatment values of study formulations and the reference formulation for significant differences by means of the Friedman analysis of variance with an agreed significance level of p = 0.05. Subsequently, we tested the differences between the log_10_ reduction factors from each study formulation and the appropriate values of the reference for significance by a nonparametric noninferiority test according to Hodges-Lehmann. We rejected inferiority of a study formulation and assumed noninferiority if the Hodges-Lehmann upper 97.5% confidence limits for the differences in log_10_ bacterial reductions between study formulations and reference treatment were smaller than the agreed inferiority margin of 0.75 log_10_ (immediate effect) or 0.85 log_10_ (3-hour effect). We set the level of significance at p = 0.025 (1-sided). Furthermore, we used the Wilcoxon matched-pairs, signed-ranks test to test for a suspected sustained effect at p = 0.01 (1-sided) if—as concluded from a higher mean log_10_ reduction—a study formulation was suspected to be more efficacious than the reference antisepsis procedure 3 hours after antisepsis.

## Results

We observed no significant differences between the means of the log_10_ pretreatment bacterial counts for the immediate and 3-hour efficacy tests. Hence, the baseline for each study formulation can be considered equivalent.

Overall, immediate effects were comparable to that of the reference alcohol of EN 12791; typical magnitude was mean log_10_ reductions of >2.00 (Table). Each modified formulation was even more effective than the reference alcohol immediately after hand antisepsis. Each modified formulation also met the 3-hour efficacy requirements of EN 12791. The mean log_10_ bacterial reduction of the formulation based on isopropanol was greater by 0.15 log_10_ than that of the reference alcohol, but this difference was not significant (p = 0.01 by Wilcoxon matched-pairs signed-ranks test), so sustained efficacy cannot be confirmed.

**Table:** Immediate and 3-hour effects after 3-minute application of modified WHO formulations compared with 3-minute applications of reference surgical hand antiseptic treatment according to European Norm 12791

Formulation	Immediate effect			3-hour effect	
	Mean log_10_ reduction	Hodges-Lehmann upper 97.5% confidence limit†		Mean log_10_ reduction	Hodges-Lehmann upper 97.5% confidence limit‡
WHO I modified with ethanol	2.99 ± 1.03	0.03 n.i.		1.95 ± 0.97	0.55 n.i.
WHO II modified with isopropanol	2.95 ± 1.03	0.23 n.i.		2.17 ± 1·48	0.47 n.i.
Reference	2.64 ± 0.93	Not applicable		2.02 ± 1.06	Not applicable

*n = 24 study participants. n.i., noninferior versus reference; WHO, World Health Organization. †With an agreed inferiority margin of 0.75 log_10_. ‡With an agreed inferiority margin of 0.85 log_10_.

## Discussion

The COVID-19 pandemic has led to scarcity of commercial hand antisepsis agents, and healthcare institutions worldwide are seeking alternatives. Since the end of February 2020, pharmacies in Europe have been producing the WHO-recommended formulations either for sale or as donations for personal use by the general population or use in healthcare settings. Use of hygienic hand preparations made with the original WHO-recommended formulations might be justifiable to prevent infection or transmission of pathogens outside patient care. However, to be approved in Europe, preparations for hygienic hand antisepsis used in healthcare facilities must meet the bactericidal efficacy requirements of EN 1500 (*5*) under practical use conditions. Both WHO-recommended formulations failed the EN 1500 requirements with use of 3 mL for 30 seconds, the common duration of application in hospitals in Europe (*4*). Sufficient bactericidal efficacy could be achieved with the original WHO-recommended formulation with 6 mL in 60 seconds or with 3 mL in 30 seconds when modified formulations with increased alcohol concentrations of 80% wt/wt ethanol or 75% wt/wt isopropanol were used (*4*). In general, a shortening of the necessary exposure time may help medical personnel comply with hand hygiene standards. A recent study (A. Kratzel et al., unpub. data, https://www.biorxiv.org/content/10.1101/2020.03.10.986711v1) showed that severe acute respiratory coronavirus 2 can be inactivated within 30 seconds by both WHO-recommended formulations but also by modifications as proposed by us in 2013 (*8*) or used by Allegranzi et al. (*10*) in a before–after intervention cohort study.

The use of WHO-recommended formulations in hospitals, including for surgical hand preparation, is paramount despite the lack of commercial agents. In Europe, before a product is allowed to be used for surgical hand preparation, its efficacy must be evaluated in the laboratory on the hands of volunteers according to EN 12791 (*3*), the most stringent available in vivo test method for efficacy testing. This testing ensures that results are generated under controlled conditions but also under as near as possible practical in vivo conditions. The bacterial reduction is measured directly after hand antisepsis on one hand (immediate effect) and after 3 hours on the other (gloved) hand (3-hour effect). According to the requirements of the norm, a formulation shall not be significantly less efficacious than a reference procedure at both times (i.e., immediately and 3 hours after application). The 2009 WHO guideline reported that WHO I did not pass EN 12791 under 2 laboratory testing conditions and WHO II under 1 of 2 laboratory testing conditions (*1*). Even prolonging the duration of application to 5 minutes, the longest duration allowed by EN 12791, did not achieve a favorable outcome for the original WHO formulations (*2*,*6*). Increasing the alcohol concentration of both formulations by ≈5% (by changing to weight percentage concentrations) rendered the immediate effect of the 2 formulations noninferior to the reference; unfortunately, the 3-hour effect was still significantly less effective than the reference alcohol (*6*). The reason for these results was attributed to the high concentration of glycerol (1.45% vol/vol). Although the 3-hour effects of each formulation with reduced glycerol content (0.725%) were rendered noninferior to the reference, glycerol-free preparations were even more effective than reference EN 12791. We have been able to show how the WHO formulations can be improved to meet the European standards; however, our proposals have not yet been endorsed. One of the arguments given was the lack of data on acceptance and tolerability for the modified formulations. Another argument was the necessary application duration of 5 minutes for surgical hand preparation, which does not correspond with common practice.

Frequent use of alcohol-based hand rubs can cause skin dryness unless emollients or humectants such as glycerol are added to the formulation. A recent study (*9*) evaluated the skin tolerability of healthcare workers to the original WHO formulation containing 1.45% glycerol against 3 other concentrations (0%, 0.5%, and 0.75%) of glycerol in a tropical climate healthcare setting. Dermal application of glycerol, a trihydroxy alcohol, increases the endogenous delivery of glycerol with improvement of stratum corneum hydration, skin barrier function, and mechanical properties. It also inhibits stratum corneum lipid phase transition, protection against irritating stimuli, and enhancement of desmosomal degradation (*11*). A modified WHO formulation containing only 0.5% glycerol leads to better ratings of skin tolerance than the original formulation and may therefore offer the best balance between skin tolerance and antimicrobial efficacy. In addition, it is useful to have the same alcohol-based hand rub formulation in the surgical setting and in other medical settings, especially if products are scarce. Because glycerol availability is also critical during the current pandemic, lowering the glycerol concentration might improve availability of these alcohol-based formulations in areas with limited supplies, such as developing countries.

In this study, we were able to show, once again, that the effect on the resident skin flora of the original WHO formulations can be improved if the concentration of the alcohols is increased by using weight instead of volume percentage. In addition, by further reducing the glycerol content from 1.45% to 0.725% or to 0.50%, the 3-hour effects of each formulation can be improved to such an extent that the requirement of the European test standard is already ensured after 3 minutes of application. Although the criteria for use as a product regulated by the US Food and Drug Administration differ from the EN requirements, these results could also be of interest to US healthcare providers. Reductions achieved with the modified formulations were >1 log_10_ step higher than those achieved with the original WHO-recommended formulations when applied for 3 minutes for both immediate and 3-hour effects (Figure).

**Figure:**
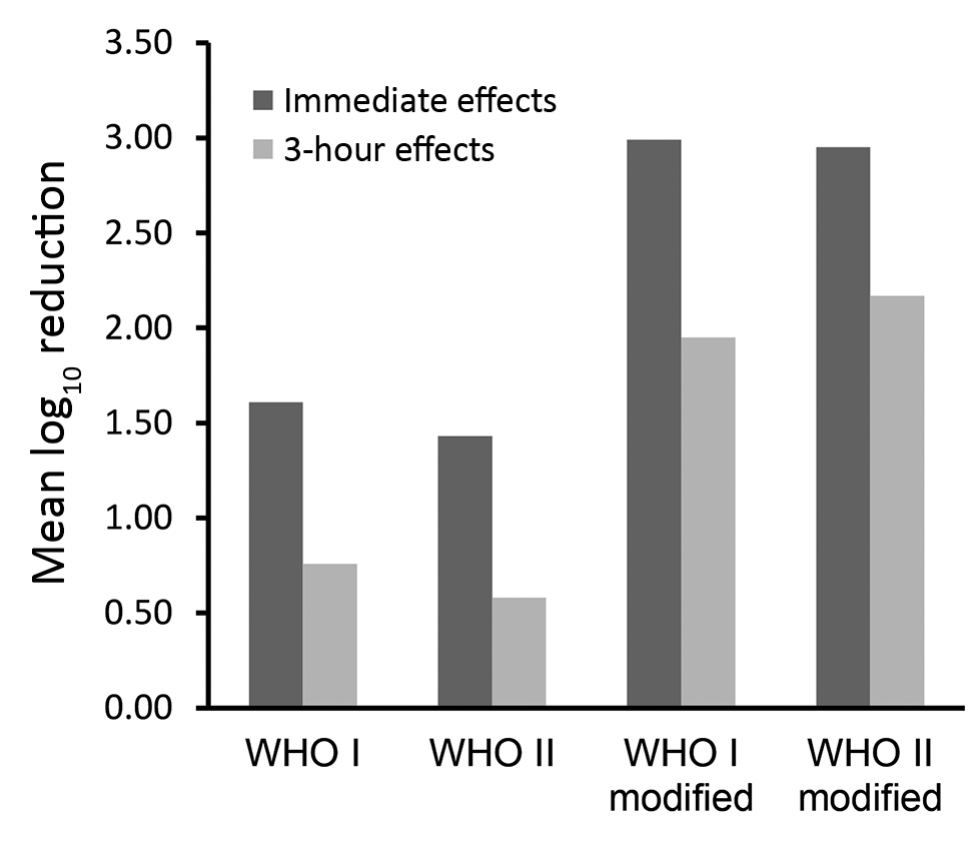
Comparison of the immediate and 3-hour effects of the WHO-recommended (WHO I and WHO II) and modified (WHO I modified and WHO II modified) formulations among 24 volunteers after a 3-minute surgical hand preparation according to European Norm 12791 (*3*). WHO I: ethanol 80% vol/vol + glycerol 1.45% vol/vol + hydrogen peroxide 0.125% vol/vol. WHO II: isopropanol 75% vol/vol + glycerol 1.45% vol/vol + hydrogen peroxide 0.125% vol/vol. WHO I modified: ethanol 80% wt/wt + glycerol 0.5% vol/vol + hydrogen peroxide 0.125% vol/vol. WHO II modified: isopropanol 75% wt/wt + glycerol 0.5% vol/vol + hydrogen peroxide 0.125% vol/vol. WHO, World Health Organization.

On the basis of these results and considering the current situation, we believe that the original WHO formulations should be urgently reconsidered. We therefore recommend a modification of the WHO I formulation with 80% wt/wt ethanol, 0.125% vol/vol hydrogen peroxide, and 0.50% vol/vol glycerol and a modification of the WHO II formulation with 75% wt/wt isopropanol, 0.125% vol/vol hydrogen peroxide, and 0.50% vol/vol glycerol.
